# Clean technology cost projections: investment and levelized costs of solar, wind, battery, and hydrogen

**DOI:** 10.1038/s41597-025-05951-4

**Published:** 2025-10-22

**Authors:** Hadi Vatankhah Ghadim, Jannik Haas, Christian Breyer, Hans Christian Gils, Philip Odonkor, E. Grant Read, Mengzhu Xiao, Rebecca Peer

**Affiliations:** 1 Sustainable Energy Research Group (SERG), Department of Civil and Natural Resources Engineering, University of Canterbury, Ōtautahi Christchurch, Aotearoa, New Zealand; 2 School of Energy Systems, LUT University, 53850 Lappeenranta, Finland; 3 German Aerospace Center (DLR), Institute of Networked Energy Systems, Curiestraße 4, 70563 Stuttgart, Germany; 4 Department of Systems and Enterprises, Charles V. Schaefer, Jr. School of Engineering and Science, Stevens Institute of Technology, Hoboken, NJ 07030 USA; 5 International Renewable Energy Agency, Innovation and Technology Center, Willy-Brandt-Allee 20, 53113 Bonn, Germany

**Keywords:** Energy modelling, Renewable energy, Energy economics, Energy infrastructure

## Abstract

Reliable cost projection data is critical for energy system modelling, guiding policy and investment decisions that underpin the global energy transition. In this work, we compile and standardise a broad dataset from over 110 existing regional and global studies to provide an organised and spatio-temporally granular dataset of cost projections for major clean energy technologies. The dataset covers Capital Expenditures (CAPEX) and Levelised Costs of Electricity or Hydrogen (LCOE or LCOH) for utility-scale and rooftop photovoltaics, onshore and offshore wind power, grid-scale Li-ion batteries, concentrated solar thermal power, and large-scale alkaline and PEM electrolysers. The data span national, continental, and global scales, with annual granularity through 2050 and metadata for source type and region. Values under various scenarios are provided to enable risk and uncertainty assessments. This resource supports scenario modelling, investment planning, policy design, and benchmarking in the context of decarbonisation pathways. While the data are drawn entirely from existing sources, the novelty lies in the structured harmonisation, metadata processing, and comprehensive coverage, making it suitable for techno-economic evaluation and robust energy system modelling.

## Background & Summary

Energy system models are crucial for helping policymakers, researchers, and industry leaders figure out the best path to a sustainable energy future^1,2^. However, these models rely heavily on cost projections for different energy technologies^3^. Recent studies have shown that many of these projections remain overly pessimistic, especially for rapidly advancing technologies such as solar PV and batteries^4^. This can bias scenario outcomes, misguide infrastructure planning, and potentially slow down the clean energy transition.

Access to well-organised and relevant cost data is essential for energy system modellers^5^. They guide energy system planning analyses to select the most efficient and cost-effective technologies^6^, ensuring that future energy systems are designed to achieve the best possible balance between cost, performance, and sustainability^7^. Previous studies have made valuable contributions by compiling renewable electricity generation technology cost data up to 2018^8^, helping lay the groundwork for modelling efforts. However, as the energy sector continues to evolve rapidly, there is a growing need for more current and consistent data^9^. Without it, modelling outcomes can be distorted and potentially affect budget planning and policy decisions. Inaccurate or outdated inputs risk steering investments toward less effective technologies^10^, which may ultimately hinder the pace of the clean energy transition^11^ and delay the achievement of climate goals^12^.

**Fig. 1:**
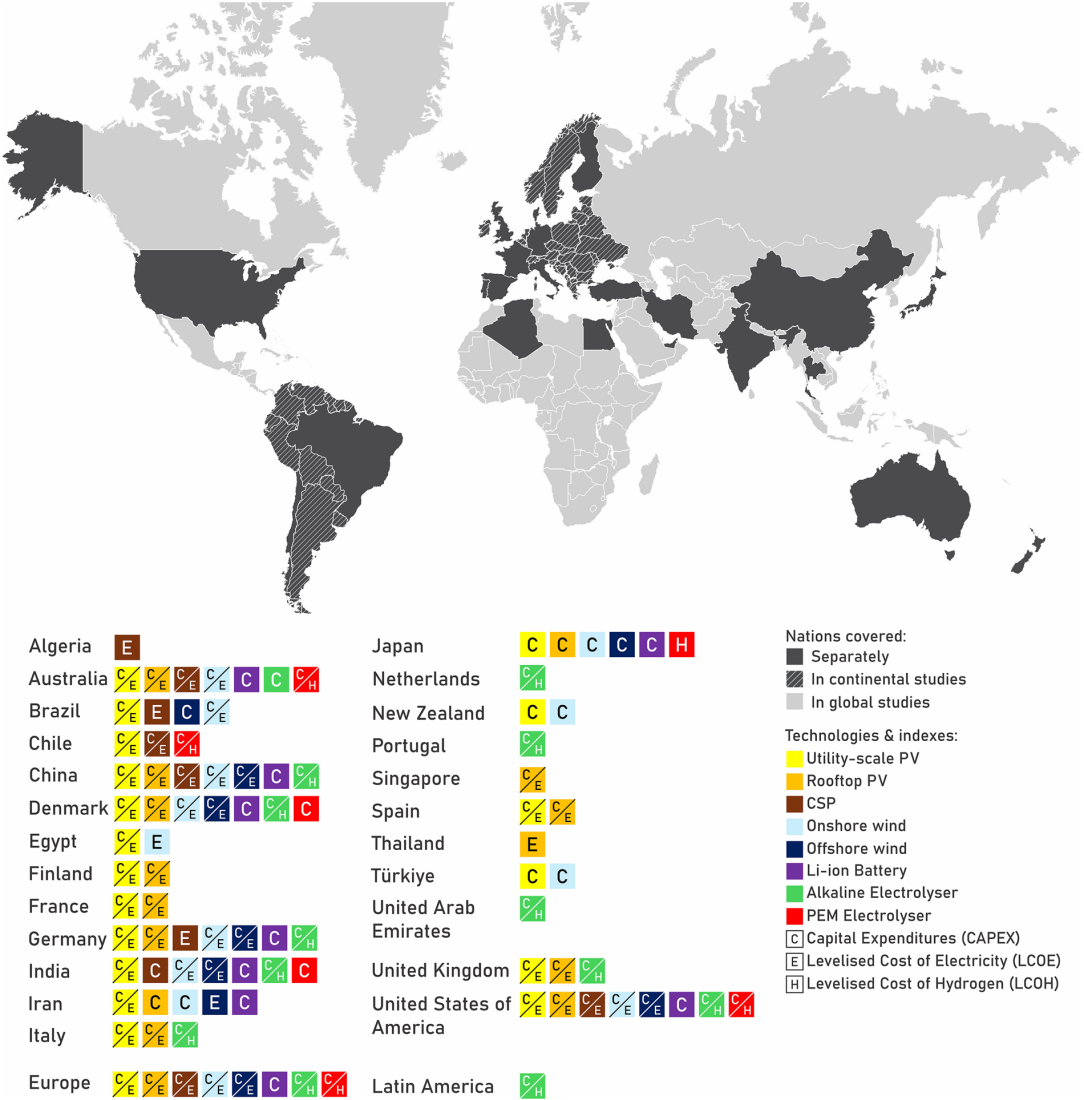
Geographical regions covered in the compiled data provided in this study, and technology diversity in the cost projections provided for each region.

This work presents a harmonised compilation of cost projections for key clean energy and emerging technologies. We focus on studies and reports published since 2020, as this period reflects the most up-to-date assumptions around technology learning rates, market dynamics, and policy contexts. Additionally, earlier studies have been comprehensively synthesised in^8^, reducing redundancy and allowing us to build on that foundation with more recent insights. Unlike primary datasets that involve new data collection or measurements, this dataset is entirely built on secondary data from peer-reviewed and institutional sources. Its value lies in the breadth of sources, temporal and spatial resolution, systematic cleaning and standardisation, and comprehensive tagging with metadata. We included:

- Utility-scale photovoltaics (PV) with installed capacity more than 1 MW, and rooftop PV (5–50 kW-scale), onshore and offshore wind power (grid-scale), stationary Li-ion batteries (grid-scale), Concentrated Solar Thermal Power (CSP) plants, Alkaline (ALK) and Proton Exchange Membrane (PEM) electrolysers (units with capacity of at least 1 MW_e_).
- Two main cost indicators, Capital Expenditures (CAPEX) and Levelised Cost of Electricity/Hydrogen (LCOE/H)
- Data from diverse regions on national, continental, and global scales (depicted in Fig. 1).

This selection of technologies is grounded in both relevance and comparability across studies. Utility-scale solar and wind power are now the lowest-cost sources of additional clean generation in many regions, with cost projections driving investment decisions and policy planning. With the rising shares of PV being installed in the residential sector, rooftop PV systems are resulting in lower electricity costs than the grid electricity prices^13^. CSP plants are included due to their ability to deliver dispatchable renewable electricity using integrated thermal energy storage, making them a valuable complement to variable renewables in regions with high Direct Normal Irradiance (DNI) such as North Africa, the Middle East, Australia, and the southwestern United States^14,15^. Li-ion stationary battery storage technologies are increasingly being deployed and analysed alongside variable renewable energy due to their capability in playing various roles in system operation, such as energy arbitrage, primary/secondary/tertiary responses, congestion management, and demand-side management^16,17^. Clean hydrogen has an emerging role as a seasonal power balancing option and energy vector to decarbonise hard-to-abate sectors^18,19^. Clean hydrogen is also one of the core elements of the future Power-to-X economy^20^, with an expected demand of around 61 PWh_LHV_ of hydrogen^21^.

While technologies such as geothermal, bio-energy, and nuclear power also contribute to clean energy transitions, they were excluded from this dataset due to 1) the limited availability of high-resolution, forward-looking cost projection data across as many scenarios and regions as is available for the four selected technologies; 2) their lower relevance in many decarbonisation pathways, especially in smaller or islanded systems; and 3) the complexity of comparing their cost structures directly, given their high site specificity and non-standardised configurations^22,23^. Some of the data reported here are also used in related publications^24,25^, which examine the intrinsic over-pessimism in cost projections of clean electricity and green hydrogen production technologies.

This database is intended to be useful in developing and implementing strategies for transitioning to renewable energy systems, performing economic analyses comparing the costs and benefits of different renewable energy technologies in the context of a defined geographical region, and evaluating the potential market development trends and future costs of renewable energy technologies. Specifically, the dataset enables:

- **Techno-economic scenario modelling**: The standardised cost trajectories across key technologies allow planners and modellers to construct or calibrate future energy system scenarios, particularly for Integrated Resource Plans (IRPs), national energy strategies, and net-zero roadmaps.
- **Investment and policy prioritisation**: By providing projections from over 100 studies, the database helps identify where and when specific technologies become cost-competitive. This is critical for determining optimal timing for public or private investments and for designing targeted policy incentives.
- **Risk and uncertainty assessment**: The inclusion of minimum, average, and maximum cost projections enables users to assess the range of potential outcomes and uncertainties, supporting robust decision-making under uncertainty (e.g., via stochastic modelling or sensitivity analyses).
- **Technology benchmarking**: The database allows users to benchmark national or regional assumptions (e.g., in power system models or hydrogen strategies) against a global range of published projections, identifying whether they are aligned with, optimistic about, or lagging international trends.
- **Cross-sectoral integration analysis**: In particular, the inclusion of battery storage and renewable hydrogen supports modelling of demand-side flexibility, sector coupling, and deep decarbonisation pathways involving power-to-gas (e.g., hydrogen), power-to-heat, and power-to-liquid fuels, often referred to as “power-to-x” solutions.

These functionalities are made possible by consistent formatting, temporal granularity (annual data to 2050), and metadata inclusion (e.g., region, source type, and publication year).

## Methods

**Fig. 2:**
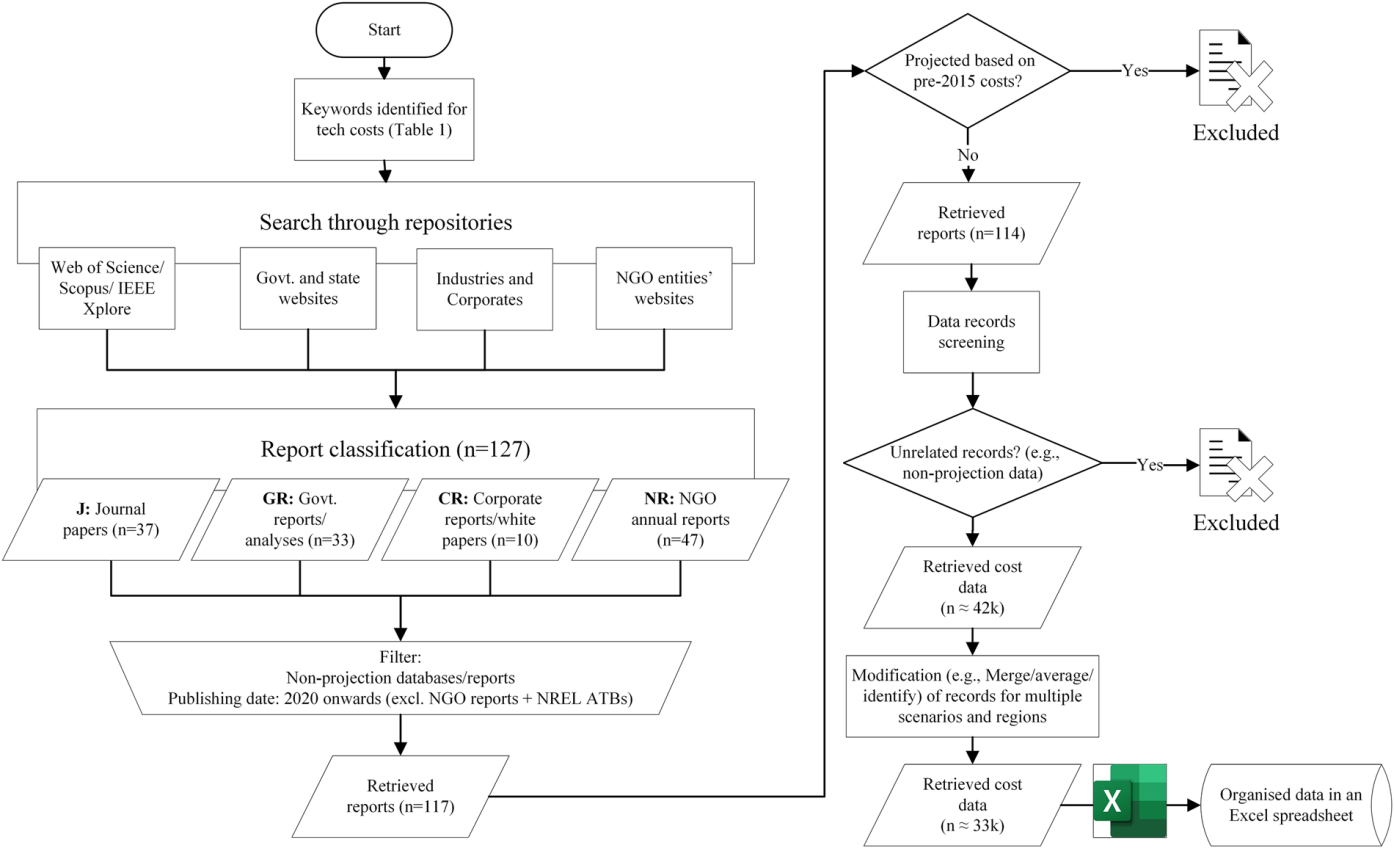
Workflow for the compilation of energy technology cost data.

Figure 2 shows the process for collecting, analysing, and aggregating data for this work. After identifying the necessary keywords (available in Table 1), we searched for published articles within different academic repositories such as Web of Science, Scopus, and IEEE Xplore (for journal papers), governmental and state-funded institutes’ websites (for reports), industries and corporates (for white papers), and NGO entities (for annual outlooks). We then classified those reports into four categories: (1) academic journal papers, (2) governmental update reports, (3) commercial/industrial white papers, and 4) NGO annual outlooks. At this stage, we were considering over 120 analyses. The next step included filtering the reports based on two criteria: (1) reports must include at least one set of projection modelling results for each scenario assessed, and (2) they must have been published after 2020. We have applied an exception to the second criterion for the Annual Technology Baseline (ATB) reports of the National Renewable Energy Laboratory (NREL) of the United States (U.S.), the International Energy Agency’s (IEA) World Energy Outlooks (WEO), renewables cost projections for Germany from the Fraunhofer Institute for Solar Energy (ISE) Systems, European Technology and Innovation Platform updates for Photovoltaics (ETIP PV), and generation technology cost projections for Australia from the Commonwealth Scientific and Industrial Research Organisation (CSIRO). These exceptions were made because these reports provide frequent updates on their projections, enabling a mechanism to monitor and record the changes in projection trendlines for major renewable electricity technologies (solar PV, onshore wind power, and offshore wind power).

**Table 1:** Selected keywords for repository search in this study.

For Renewable Electricity Technologies	For Electrolysis Technologies
**Cost Indicators**	
LCOE	LCOH
Levelised cost of electricity	Levelised cost of hydrogen
Cost per MWh	Cost per kg of hydrogen
Levelised cost of delivered electricity	Capital expenditures
Capital expenditure	Cost per kW_el_
Cost per kW_p_	
**Technologies**	
Utility-scale solar PV	AEL
Large-scale solar PV	Alkaline electrolysis
Rooftop PV	AEC
Residential rooftop solar PV	Alkaline electrolysis cells
Commercial and Industrial rooftop solar PV	PEM
Concentrated solar thermal power	Proton exchange membrane
Onshore wind power	Polymer electrolyte membrane
Offshore wind power	
Stationary battery energy storage	

The filtering process resulted in 117 suitable reports. We manually reviewed the data records within each report and excluded those that used cost assumptions from studies earlier than 2015 for their projection modelling (to ensure we captured the potential impact from the Paris Agreement). We then screened the remaining 114 reports to collect the necessary data for our study, which included investment costs and levelised costs of electricity and hydrogen. We have used the projection data from those reports as our inputs. For the rest, we have labelled them as “unrelated records”. Unrelated records include the assumptions made to obtain projections, such as discount rates, learning rates, and technology lifetime. Finally, data points that significantly deviated from the mean and could not be explained by regional or technological differences were also excluded from the final dataset of the current paper.

The remaining data went through a “processing” stage. To ensure consistency and comparability across sources, several processing steps were applied to the collected cost projection data:

- **Geographical Averaging**: In many cases, individual studies reported separate cost projections for multiple regions within a single country (for example, different solar irradiance zones across the U.S. in the NREL ATB). Where appropriate, we averaged these regional values to obtain a single, country-level cost projection. This approach aligns with our assumption that each country is treated as a single node in the context of long-term energy system modelling. Similarly, for European Union-level data, such as those from the ETIP PV reports, we averaged values reported for several representative cities across different parts of Europe (e.g., London, Munich, Toulouse, Rome, Malaga, Helsinki) to generate an average and a cost range for Europe as a whole.
- **Standardising Component Inclusions**: In some older datasets, certain technology cost components were not fully accounted for. For example, in the NREL ATB utility-scale PV CAPEX data from 2015 to 2019, the CAPEX was reported in USD/kW_dc_, representing the solar module costs only. However, the PV CAPEX reported in studies from 2020 onwards were in USD/kW_ac_, including the system output cost. To harmonise these datasets and enable meaningful comparison over time, we applied a correction factor of 1.34 to the older NREL values, following the guidance provided in the NREL ATB documentation^26^. DC/AC ratios for utility-scale PV typically fall between 1.1 and 1.5^27^. However, various references use values closer to 1.2 as a typical default^28^. While the value applied in this work lies at the higher end of the usual range, it was chosen to reflect NREL’s own standardisation practices and ensure consistency across the full time series. We acknowledge that these ratios can vary significantly depending on installation type, project location, and local regulatory frameworks. Therefore, users interested in more granular or location-specific modelling should consider adjusting this factor based on system configuration.
- **Filling Gaps and Smoothing Trends**: When a report provided only minimum and maximum cost projections, without an average or median value, we estimated the average ourselves, by averaging the minimum and maximum, to allow for consistent comparison across studies. This simple approach is used due to the absence of further statistical information of input data. This step ensured each dataset contributed consistently to the final visualisations and trend analyses.

These processing steps were necessary to unify diverse data sources into a coherent and usable format for long-term techno-economic analysis and cross-regional comparison. Overall, we used data from 114 studies^29–139^, with over 33,000 data records to compile our final cost projection database. A list of reports is available in Supplementary Table 1.

We have compiled the extracted data in an Excel workbook. The workbook contains individual sheets dedicated to each renewable electricity technology, including Utility-scale PV, Rooftop PV, Onshore wind power, Offshore wind power, CSP, stationary Li-ion batteries, Alkaline, and PEM electrolysers. For each technology, there are two cost indicators: CAPEX and LCOE projections. In addition, we have compiled both CAPEX and LCOE data for all relevant technologies from major institutional sources: NREL ATBs, IEA WEOs, CSIRO, Fraunhofer ISE, and ETIP PV reports. We have included data from reports published before 2020 in the reports from those five institutions, in contrast to the others, which contain only forward-looking projections from 2020 onwards. The following sub-sections will describe the data included from various studies and explain how we obtained and displayed the records from those reports, in special cases. Section (a) provides details on how data was acquired from academic and industrial publications. Section (b) provides further details on data processing for NREL ATB reports, and Section (c) explains how the cost data was extracted from IEA WEO reports.

### Academic studies and published reports

In this category, we either used the data provided within the text of those papers or supplementary files. We applied adjustments for variations in currency and inflation rates throughout the analysed timeframe. Cost figures were standardised to USD 2024 by multiplying costs from the studies with inflation rates derived from the Consumer Price Index (CPI) of the U.S. Bureau of Labour Statistics, and currencies were converted using average annual exchange rates for each region (Eq. 1). In the absence of numerical data, we extracted values from visual graphics of cost projections or explanations provided within the publication on the assumptions made for the projection. For electrolyser technologies, we also included information about the electricity sources assumed in their cost projections. This supports clearer comparison and tracking of the capital costs (CAPEX) and levelised cost of hydrogen (LCOH) for green hydrogen production.

### National renewable energy laboratory annual technology baseline reports (NREL ATB) 2015–2024

For NREL ATB studies^29–38^, we used the latest spreadsheets published by the NREL team. Those spreadsheets include the technical details, structural specifications, cost assumptions, and projections for the future for each of the present technologies in the United States. In earlier versions of these spreadsheets, the range of generation and storage technologies was limited. For instance, no data was included regarding various types of onshore wind power (central or distributed), offshore wind power (fixed-bottom or floating), rooftop PV (either residential, commercial, or industrial), and battery energy storage (utility-scale, commercial, residential, or coupled with solar PV). NREL ATB reports have presented different projections under three scenarios consistently: (1) Conservative: a scenario in which the technology changes little from today, and progress is driven mainly by historical investments and existing industrial learning, (2) Moderate: current levels of research innovation continue, making today’s emerging technologies more widespread and leading to steady, expected improvements in technology, and (3) Advanced: this scenario assumes breakthrough innovations (e.g., improved efficiency) and new technology architectures that are very different from today’s, accelerating transformation across the market. Where sources provided data for different classifications within one category of technology, we adopted the following approach:

- We used the highest of the costs in conservative scenarios and the lowest of the costs in advanced scenarios reporting the maximum and minimum cost ranges, respectively.
- We then averaged the rest of the costs for all the classifications to determine the midpoint (e.g., in the case of utility-scale solar PV for the year 2024, the NREL ATB dataset has 10 different classifications, each representing the cost of installation in areas with different Global Horizontal Irradiance (GHI) levels in the U.S.).
- Lastly, if the cost was the same in all three technology scenarios (conservative, moderate, and advanced), like in some older NREL databases, we used the common values.

### International energy agency (IEA) world energy outlook (WEO) cost projections 2000–2024

In the case of WEO projections^39–60^, we used LCOE data provided directly in the reports (e.g., appendices or in-text data) without performing any additional calculations, as we had limited access to the required cost assumptions and calculation processes within the IEA energy transition model. For CAPEX values, we used data in the reports when available and calculated values when data was not available following Eq. 1:

1 $$\frac{{Investment}\left({{bUSD}}_{{year}}\right)\times {CER}}{{Capacity}\left({MW}\right)}={CAPEX}\left(\frac{{{USD}}_{2024}}{{kW}}\right)$$

The investment parameter includes aggregated capital expenditures for the mentioned capacity development (in billion USD). The currency equivalence rate (CER) is the required rate for inflation adjustment rates derived from the (CPI) of the U.S. Bureau of Labor Statistics. Given the diversity across the annual WEO reports in terms of data presentation and availability we made manual adjustments to calculate CAPEX, as summarised in Table 2.

**Table 2:** Detailed processes of extracting data from IEA’s WEO reports since 2000.

WEO report years	Data issue	Adjustment applied in this work
2000–2013	Cost data for marine, geothermal, and CSP are aggregated	• CSP CAPEX was only calculated via Eq. 1 for countries where capacities of geothermal and marine were equal to zero.
2014, 2015, 2017	No available data on CAPEX for renewable technologies	• Average investment values for technologies for each timeframe (5-years) were extracted from figures within the reports. Total annual costs for installed technology capacities were determined by multiplying by the number of years in each timeframe. • Cumulative installed capacities were extracted from the “New Policies Scenario” table in the appendices. Annual net capacity additions were determined by subtracting the previous period’s existing technology capacities. • The CAPEX was calculated using Eq. 1.
2016	No available data on CAPEX for renewable technologies	• Average investment costs for 2011–2016 were extracted from report figures. • Annual net capacity additions for 2016 were determined by subtracting cumulative capacity installed in 2016 from those in 2015 from the “New Policies Scenario” table in the appendices. • For solar PV and wind, installed capacities were extracted from WEO 2013 for the year 2011. These were subtracted from the 2016 capacities to get capacity additions from 2011–2026. • The CAPEX was calculated through dividing the total investment cost from 2011–2016 by the added capacity.
2018–2019	No global reference CAPEX projection	CAPEX for solar PV, onshore, and offshore wind power are extracted from Table B.6 (under “New Policies Scenario” for WEO 2018, and under “Stated Policies Scenario” for WEO 2019) for India, China, the EU, and the U.S. and adjusted to the inflation rate. Country values were averaged to determine a global reference CAPEX projection and the highest and lowest values used to represent the range at each time point.
2020	No global reference CAPEX projection	• CAPEX values from the “Stated Policies Scenario” and “Sustainable Development Scenario” were extracted from tables in the report and adjusted for inflation.
2021–2024	No global reference CAPEX projection	• CAPEX for solar PV, onshore, and offshore wind power were extracted for India, China, the EU, and the U.S. from the appendices for the “Stated Policies Scenario” (highest estimates), “Announced Pledges Scenario”, and “Net Zero Emissions” (lowest estimates) for 2020, 2030, and 2050. • Country values were averaged to determine a global reference CAPEX projection and the highest and lowest values used to represent the range at each time point.

## Data Record

**Fig. 3:**
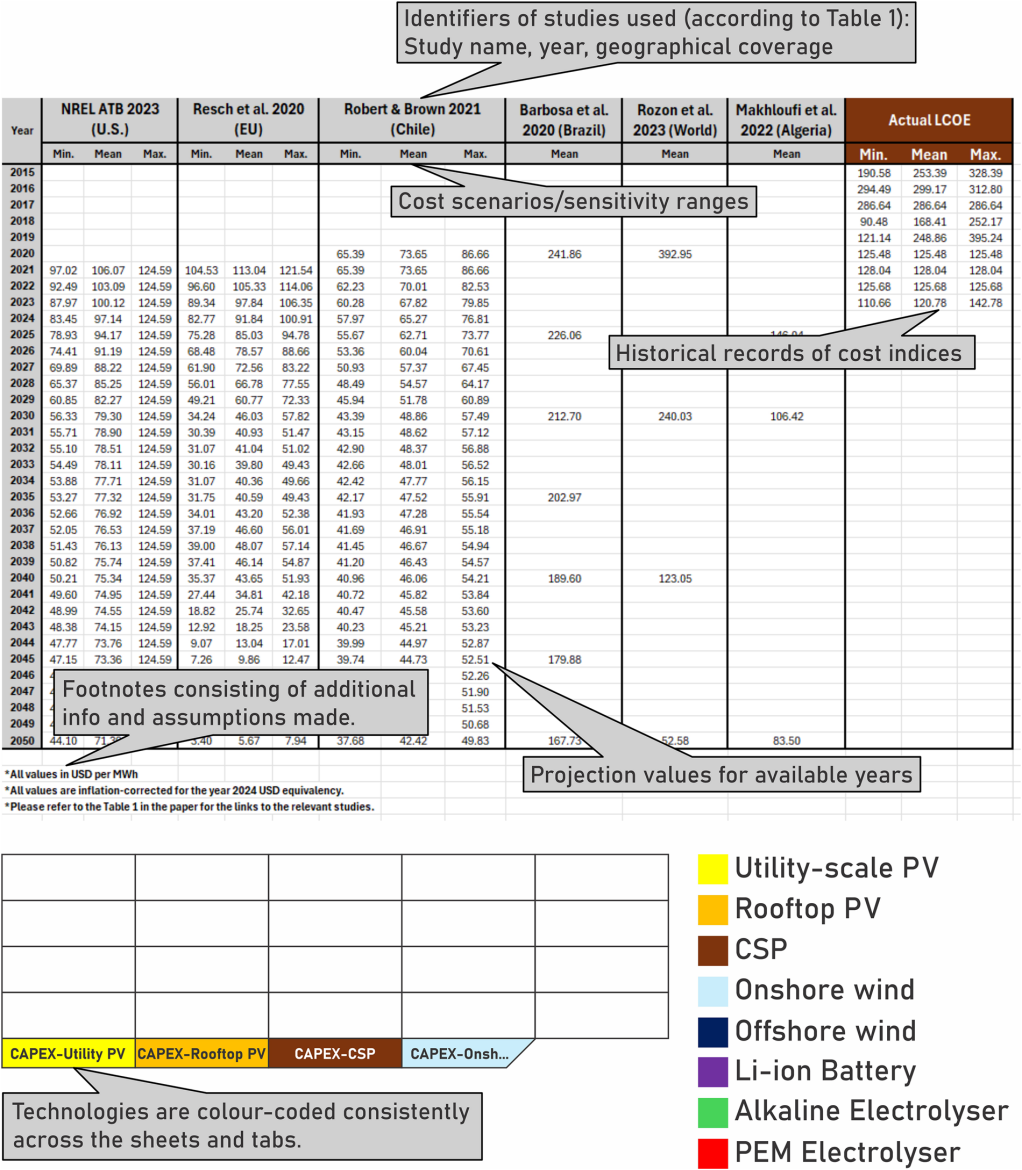
Illustrated view of the compiled workbook.

The data has been compiled into two structured Excel workbooks, available through a public Zenodo repository^140^. The first Excel spreadsheet has 21 sheets. The “Conversion rates” sheet includes all the currency conversion rates used to adjust older studies to the inflation rate. The next eight sheets include compiled projections for CAPEX of different technologies. These sheets are both named and colour-coded based on the provided legend in Fig. 3. The following seven sheets are also dedicated to the compiled LCOE projections. The last five sheets are for the NREL ATBs, IEA WEOs, CSIRO Gen-Cost, Fraunhofer ISE, and ETIP PV reports. The second spreadsheet is a machine-friendly version of the first spreadsheet, for higher readability in coding processes. This spreadsheet also follows the same structure as the first one, with differences in column titles. The column titles in each sheet include the study identifiers (refer to Supplementary Table 1), and scenario names (e.g., Breyer_et_al_(EU)_min shows the lowest projected costs available in^61^ that provides cost projections for Europe). Column titles in the last five sheets that are for the NREL ATBs, IEA WEOs, CSIRO Gen-Cost, Fraunhofer ISE, and ETIP PV reports, include the cost index, the technology, and variants for scenarios in addition to the previous title structure (e.g., OEDI_2024_(US)_Utility_PV_CAPEX_Advanced shows the projected CAPEX under the “Advanced” scenario for Utility-scale PV in^29^ that provides cost projections for the U.S.). The usage of underscores instead of space in column titles is to facilitate higher readability during coding.

For academic studies, projections are categorised as “min”, “mean”, and “max” values, reflecting the sensitivity ranges reported in each study. For institutional datasets from NREL, IEA, Fraunhofer ISE, ETIP, and CSIRO, we preserved the original scenario names provided by the sources. For example, IEA WEO had Stated Policies Scenario (STEPS), Announced Pledges Scenario (APS), Sustainable Development Scenario (SDS), and Net Zero Emissions Scenario (NZE). CSIRO reports used “450 ppm” and “550 ppm” in the 2015 report, and “2 °C” and “4 °C” in the 2017–2018 report, which were directly reflected in our spreadsheet.

For comparability across studies and years, all cost values are expressed in USD per kW (for CAPEX) or USD per MWh (for LCOE) and are inflation-adjusted to 2024 USD. Each sheet is organised by calendar year (typically 2015–2050, except for the institutional datasheets), with columns for cost values (min., mean, max., or scenario-based) from multiple sources. This layout supports a wide range of queries and analyses, including comparing mean CAPEX or LCOE values from several studies for a specific year (e.g., 2035), analysing the range (min. to max.) of projections for a single country (e.g., India, China, US), tracking long-term cost trends for a given technology across studies, and examining institutional scenario pathways (e.g., how IEA’s NZE compares to APS or STEPS). An illustrative view of a sheet is available in Fig. 3.

## Technical Validation

To ensure the compiled database is valid for use by other studies, we have gone through a series of steps to gather, clean, and organise our database. We used CAPEX and LCOE/H data for renewable electricity and green hydrogen production technologies from peer-reviewed journal papers, annual reports from reputable institutions (e.g., U.S. national laboratories), and globally recognised energy databases such as IRENA and IEA WEOs. We also incorporated a limited number of industry reports that are widely used in energy system modelling studies and have been referenced in multiple reputable academic journals. Specifically, we included:

- BloombergNEF Hydrogen Economy Outlook (2020)^62^,
- BloombergNEF New Energy Outlook (2021)^63^,
- BloombergNEF Battery Pack Prices (2023)^64^,
- British Petroleum Energy Outlook (2022)^65^,
- E Source Battery Market Forecast (2022)^66^,
- General Electric Future of Energy White Paper (2021)^141^.

These reports were selected because their methodologies are based on validated modelling approaches, extensive expert consultation, and scenario-based assessments, often comparable in rigour to academic methods. Moreover, each has been cited in at least two to three peer-reviewed journal articles, confirming their acceptance by the scholarly community. For example, the BNEF 2020 is cited in^142–144^. The BNEF 2021 report is also cited in^145^, and^146^, which the latter is an extended study based on the same model used for the report. The BP and General Electric reports have been referenced in^147,148^ and^149,150^, respectively. The consistent use of these sources across diverse studies demonstrates their reliability and relevance in modelling clean energy transitions and technology cost pathways. While it is challenging to validate the compiled data, we relied on the validation approaches published by those studies to ensure our compiled database was as validated as possible. These sources each rely on their own internal or external (e.g., scientific peer review) validation.

**Fig. 4:**
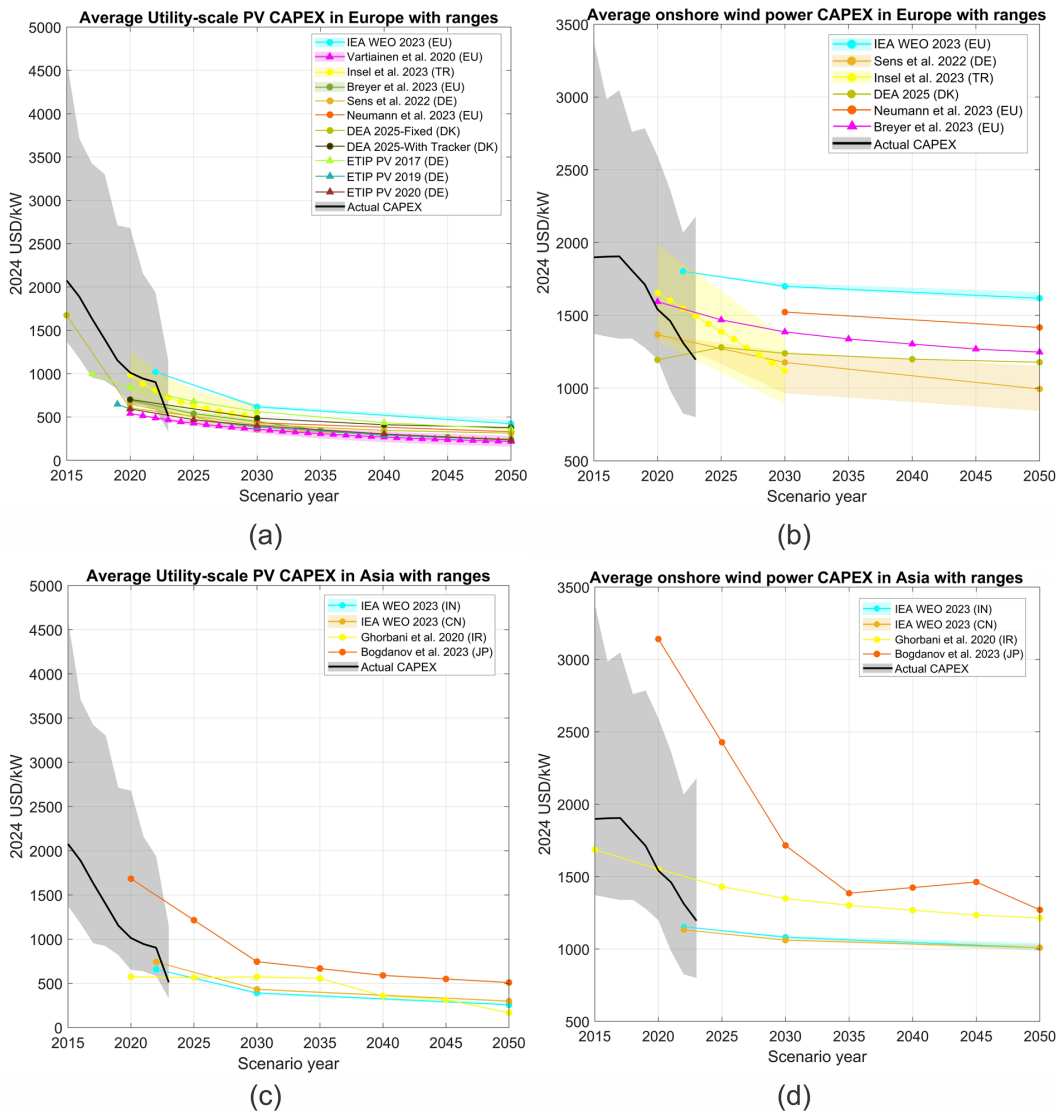
CAPEX projections from different studies for utility-scale PV and onshore wind power technologies in two regions: (**a**) utility-scale PV in Europe, (**b**) onshore wind in Europe, (**c**) utility-scale PV in Asia, and (**d**) onshore wind in Asia. The black solid line in each subplot represents the historical CAPEX trend for the corresponding technology, and the shaded grey area around it indicates the range of observed historical costs.

To ensure data validity, a thorough cross-verification process was applied wherever possible, comparing data from multiple sources within the same regions and periods. Figure 4a–d compares various continental and regional cost projections for utility-scale PV and onshore wind power technologies in Europe and Asia, respectively. The degree of consistency across multiple independent studies, alongside the alignment with established data from widely recognised sources like the IEA, provides confidence that the projection data from the selected studies for all those regions are reliable for use in further analyses. Unfortunately, we could not replicate the same comparison for the projections for other regions due to the limited number of studies available.

For other regions in the world, we found that the IEA WEOs only give future CAPEX estimates for renewable electricity technologies in four big markets: China, India, Europe, and the U.S. These estimates were carefully checked by analysing data from over 750 manufacturing plants. The analysis compared both capital and operational costs between regions. Also, it included ongoing expenses (operational costs), financial support (like government incentives), and non-cost factors such as market size, political stability, and rules about the environment, society, and governance. Because this process was so thorough, we decided it was safe to use the IEA’s cost range, from China as the lowest-cost case to the U.S. as the highest-cost case, as a guide when reviewing other studies. We found that nearly all other cost projections fall within this IEA range. This makes us more confident that the projections from other countries are reliable and suitable for future analysis. The data we provide also serves as a useful reference for anyone who wants to double-check or compare later on.

## Usage Notes

The dataset is provided in an Excel spreadsheet format with column names reflecting the column identifiers mentioned in Supplementary Table 1 and scenario titles in each study. This is to facilitate interoperability with common energy modelling tools (e.g., Excel-based models, Python scripts). Users should note that the dataset is intended primarily for forward-looking techno-economic analysis and may not fully reflect historical cost data or context-specific assumptions (e.g., local taxes, labour costs, or financing conditions). The projections are inflation-adjusted, but users are encouraged to consult original sources, referenced in Supplementary Table 1, for methodological details and assumptions behind each scenario.

One of the main limitations of this study is the lack of sufficient data for countries beyond those we found. Many regions, particularly in the developing world, have limited publicly available data on CAPEX and LCOH/E for energy technologies. Additionally, we had to rely on extracting values from visual open-access resources, particularly in reports where the underlying data and assumptions were not provided. Another challenge was limited access to resources published in languages other than English. For instance, projections for Chile were sourced from the Chilean Ministry of Energy, but the explanations were originally in Spanish, and similarly, data from Brazil were in Portuguese. While translations were made, the nuances in the original explanations could have impacted our interpretation of these projections.

## Supplementary information

Supplementary Table 1

## Data Availability

The plotting code used in Fig. 4 is also available at the same public Zenodo repository^140^ in .m file format and can be accessed with MATLAB 2023b to replicate (10.5281/ZENODO.16417026).
