# Supplementary material for: Clean technology cost projections: investment and levelized costs of solar, wind, battery, and hydrogen
Source: Sci Data. 2025 Oct 22;12:1670. doi: 10.1038/s41597-025-05951-4 (PMC12546615; doi:10.1038/s41597-025-05951-4)
Supplement: Supplementary file 1 — Supplementary Table 1 [file 41597_2025_5951_MOESM1_ESM.docx]

Supplementary Table 1: List of studies used in creating the cost database

| Reference | Datasheet column identifier | Type* | Title | Geographical scope | Published year | Considered technologies | | | | | | | | | | | | | | |
| --- | --- | --- | --- | --- | --- | --- | --- | --- | --- | --- | --- | --- | --- | --- | --- | --- | --- | --- | --- | --- |
|  |  |  |  |  |  | CAPEX | | | | | | | | Levelised cost | | | | | | |
|  |  |  |  |  |  | Utility-scale PV | Rooftop PV | CSP | Onshore wind | Offshore wind | Li-ion Battery | ALK | PEM | Utility-scale PV | Rooftop PV | CSP | Onshore wind | Offshore wind | ALK | PEM |
| (DEA, 2025) | DEA 2025 | GR | Generation of Electricity and District heating – Technology descriptions and projections for long-term energy system planning | Denmark | 2025 | ✓ | ✓ |  | ✓ | ✓ | ✓ | ✓ | ✓ | ✓ | ✓ |  | ✓ | ✓ |  |  |
| (Graham et al., 2025) | Graham et al. 2025 | GR | GenCost 2023-24 – Final report | Australia | 2025 | ✓ | ✓ | ✓ | ✓ | ✓ | ✓ | ✓ | ✓ | ✓ |  | ✓ | ✓ | ✓ |  |  |
| (OEDI, 2024) | OEDI, 2024 | GR | 2024 Annual Technology Baseline (ATB) Cost and Performance Data for Electricity Generation Technologies | U.S. | 2024 | ✓ | ✓ | ✓ | ✓ | ✓ | ✓ |  |  | ✓ | ✓ | ✓ | ✓ | ✓ |  |  |
| (IEA, 2024) | IEA 2024 | NR | World Energy Outlook 2024 | U.S., Europe, China, India | 2024 | ✓ |  |  | ✓ | ✓ | ✓ | ✓ | | ✓ |  |  | ✓ | ✓ |  |  |
| (Kost et al., 2024) | Kost et al. 2024 | NR | Levelized Cost of Electricity- Renewable Energy Technologies (Fraunhofer ISE) | Germany | 2024 |  |  |  |  |  | ✓ |  |  | ✓ | ✓ |  | ✓ | ✓ |  |  |
| (Graham et al., 2024) | Graham et al. 2024 | GR | GenCost 2023-24 – Final report | Australia | 2024 | ✓ | ✓ | ✓ | ✓ | ✓ | ✓ | ✓ | ✓ | ✓ |  | ✓ | ✓ | ✓ |  |  |
| (OEDI, 2023) | OEDI 2023 | GR | 2023 Annual Technology Baseline (ATB) Cost and Performance Data for Electricity Generation Technologies | U.S. | 2023 | ✓ | ✓ | ✓ | ✓ | ✓ | ✓ |  |  | ✓ | ✓ | ✓ | ✓ | ✓ |  |  |
| (IEA, 2023) | IEA 2023 | NR | World Energy Outlook 2023 | U.S., Europe, China, India | 2023 | ✓ |  |  | ✓ | ✓ | ✓ | ✓ | | ✓ |  |  | ✓ | ✓ |  |  |
| (Graham et al., 2023) | Graham et al. 2023 | GR | GenCost 2022-23 – Final report | Australia | 2023 | ✓ | ✓ | ✓ | ✓ | ✓ | ✓ | ✓ | ✓ | ✓ |  |  | ✓ | ✓ |  |  |
| (Bogdanov et al., 2023) | Bogdanov et al. 2023 | J | Energy transition for Japan: Pathways towards a 100% renewable energy system in 2050 | Japan | 2023 | ✓ | ✓ |  | ✓ | ✓ | ✓ |  |  |  |  |  |  |  |  |  |
| (Mandys et al., 2023) | Mandys et al. 2023 | J | Levelized cost estimates of solar photovoltaic electricity in the United Kingdom until 2035 | United Kingdom | 2023 |  |  |  |  |  |  |  |  | ✓ | ✓ |  |  |  |  |  |
| (Neumann et al., 2023) | Neumann et al. 2023 | J | The potential role of a hydrogen network in Europe | Europe | 2023 | ✓ | ✓ |  | ✓ | ✓ | ✓ |  |  |  |  |  |  |  |  |  |
| (Breyer et al., 2023) | Breyer et al. 2023 | J | Reflecting the energy transition from a European perspective and in the global context—Relevance of solar photovoltaics benchmarking two ambitious scenarios | Europe | 2023 | ✓ | ✓ | ✓ | ✓ | ✓ | ✓ |  |  |  |  |  |  |  |  |  |
| (Transpower, 2023) | Transpower 2023 | GR | TPM determination: BBC Assumptions Book – Version 1.1 | New Zealand | 2023 | ✓ |  |  | ✓ |  |  |  |  |  |  |  |  |  |  |  |
| (OEDI, 2022) | OEDI 2022 | GR | 2022 Annual Technology Baseline (ATB) Cost and Performance Data for Electricity Generation Technologies | U.S. | 2022 | ✓ | ✓ | ✓ | ✓ | ✓ | ✓ |  |  | ✓ | ✓ | ✓ | ✓ | ✓ |  |  |
| (IEA, 2022b) | IEA 2022 | NR | World Energy Outlook 2022 | U.S., Europe, China, India | 2022 | ✓ |  |  | ✓ | ✓ | ✓ | ✓ | | ✓ |  |  | ✓ | ✓ |  |  |
| (Sens et al., 2022) | Sens et al. 2022 | J | Capital expenditure and levelized cost of electricity of photovoltaic plants and wind turbines – Development by 2050 | Germany | 2022 | ✓ |  |  | ✓ | ✓ |  |  |  | ✓ |  |  | ✓ | ✓ |  |  |
| (Insel et al., 2022) | Insel et al. 2022 | J | Assessment and determination of 2030 onshore wind and solar PV energy targets of Türkiye considering several investment and cost scenarios | Türkiye | 2022 | ✓ |  |  | ✓ |  |  |  |  |  |  |  |  |  |  |  |
| (Gulagi et al., 2022) | Gulagi et al. 2022 | J | The role of renewables for rapid transitioning of the power sector across states in India | India | 2022 |  |  | ✓ |  |  |  |  |  |  |  |  |  |  |  |  |
| (Makhloufi et al., 2022) | Makhloufi et al. 2022 | J | Multi-objective cuckoo search algorithm for optimized pathways for 75 % renewable electricity mix by 2050 in Algeria | Algeria | 2022 |  |  |  |  |  |  |  |  |  |  | ✓ |  |  |  |  |
| (Gandhi et al., 2022) | Gandhi et al. 2022 | J | Catching the hydrogen train: economics-driven green hydrogen adoption potential in the United Arab Emirates | U.A.E. | 2022 |  |  |  |  |  |  | ✓ | ✓ |  |  |  |  |  | ✓ | ✓ |
| (George et al., 2022) | George et al. 2022 | J | Is blue hydrogen a bridging technology? - The limits of a CO2 price and the role of state-induced price components for green hydrogen production in Germany | Germany | 2022 |  |  |  |  |  |  | ✓ | ✓ |  |  |  |  |  | ✓ | ✓ |
| (Janssen et al., 2022) | Janssen et al. 2022 | J | Country-specific cost projections for renewable hydrogen production through off-grid electricity systems | Denmark, Portugal, Netherlands | 2022 |  |  |  |  |  |  | ✓ |  |  |  |  |  |  | ✓ |  |
| (Gilmore et al., 2022) | Gilmore et al. 2022 | J | Clean energy futures: An Australian based foresight study | Australia | 2022 |  |  |  |  |  |  |  |  |  |  |  |  |  |  | ✓ |
| (Graham et al., 2022) | Graham et al. 2022 | GR | GenCost 2021-22 – Final report | Australia | 2022 | ✓ | ✓ | ✓ | ✓ | ✓ | ✓ | ✓ | ✓ | ✓ |  | ✓ | ✓ | ✓ |  |  |
| (IEA, 2021a) | IEA 2021 – H2 | NR | Hydrogen in Latin America: From near-term opportunities to large-scale deployment | Latin America, World | 2021 |  |  |  |  |  |  | ✓ | ✓ |  |  |  |  |  | ✓ |  |
| (OEDI, 2021) | OEDI 2021 | GR | 2021 Annual Technology Baseline (ATB) Cost and Performance Data for Electricity Generation | U.S. | 2021 | ✓ | ✓ | ✓ | ✓ | ✓ | ✓ |  |  | ✓ | ✓ | ✓ | ✓ | ✓ |  |  |
| (IEA, 2021b) | IEA 2021 | NR | World Energy Outlook 2021 | U.S., Europe, China, India | 2021 | ✓ |  |  | ✓ | ✓ | ✓ | ✓ | | ✓ |  |  | ✓ | ✓ |  |  |
| (Kost et al., 2021) | Kost et al. 2021 | NR | Levelized Cost of Electricity- Renewable Energy Technologies (Fraunhofer ISE) | Germany | 2021 |  |  |  |  |  | ✓ |  |  | ✓ | ✓ |  | ✓ | ✓ |  |  |
| (General Electric, 2021) | General Electric 2021 | CR | Accelerated growth of renewables and gas power can rapidly change the trajectory on climate change | Egypt, Brazil | 2021 | ✓ |  |  |  |  |  |  |  | ✓ |  |  | ✓ |  |  |  |
| (Robert & Brown, 2021) | Robert & Brown 2021 | GR | Proyecciones de costos inversión y LCOE | Chile | 2021 | ✓ |  | ✓ | ✓ |  | ✓ |  |  | ✓ |  | ✓ |  |  |  |  |
| (Lu et al., 2021) | Lu et al. 2021 | J | Combined solar power and storage as cost-competitive and grid-compatible supply for China's future carbon-neutral electricity system | China | 2021 |  | ✓ |  |  |  |  |  |  |  | ✓ |  |  |  |  |  |
| (Graham et al., 2021) | Graham et al. 2021 | GR | GenCost 2020-21 – Final report | Australia | 2021 | ✓ | ✓ | ✓ | ✓ | ✓ | ✓ | ✓ | ✓ | ✓ |  | ✓ | ✓ |  |  |  |
| (AEMO, 2021) | AEMO 2021 | GR | 2021 Costs and Technical Parameter Review | Australia | 2021 |  |  | ✓ |  |  |  |  |  |  |  |  |  |  |  |  |
| (Zhang et al., 2021) | Zhang et al. 2021 | J | Study of China's Optimal Concentrated Solar Power Development Path to 2050 | China | 2021 |  |  | ✓ |  |  |  |  |  |  |  |  |  |  |  |  |
| (UK Department of Business Energy & Industrial, 2021) | UK BEIS 2021 | GR | Hydrogen Production Costs 2021 | United Kingdom | 2021 |  |  |  |  |  |  | ✓ | ✓ |  |  |  |  |  | ✓ | ✓ |
| (WEC, 2021) | WEC 2021 | NR | Decarbonised hydrogen imports into the European Union: challenges and opportunities | Europe | 2021 |  |  |  |  |  |  |  | ✓ |  |  |  |  |  |  |  |
| (Repenning et al., 2021) | Repenning et al. 2021 | GR | Projektionsbericht 2021 für Deutschland | Germany | 2021 |  |  | ✓ |  |  |  |  |  |  |  |  |  |  |  |  |
| (Graham et al., 2020) | Graham et al. 2020 | GR | GenCost 2019-20 – Final report | Australia | 2020 | ✓ | ✓ | ✓ | ✓ | ✓ | ✓ |  |  | ✓ |  | ✓ | ✓ |  |  |  |
| (MME & EPE, 2020) | MME & EPE 2020 | GR | Plano Nacional de Energia 2050 | Brazil | 2020 | ✓ |  |  | ✓ | ✓ |  |  |  |  |  |  |  |  |  |  |
| (Barbosa et al., 2020) | Barbosa et al. 2020 | J | When is the sun going to shine for the Brazilian energy sector? A story of how modelling affects solar electricity | Brazil | 2020 |  |  |  |  |  |  |  |  |  |  | ✓ |  |  |  |  |
| (Ghorbani et al., 2020) | Ghorbani et al. 2020 | J | Assessment of a cost-optimal power system fully based on renewable energy for Iran by 2050 – Achieving zero greenhouse gas emissions and overcoming the water crisis | Iran | 2020 | ✓ | ✓ |  | ✓ |  | ✓ |  |  | ✓ |  |  |  | ✓ |  |  |
| (ETIP, 2020) | ETIP 2020 | NR | Fact sheets about photovoltaics | Europe (Germany, France, Spain, United Kingdom, Italy, Finland) | 2020 | ✓ | ✓ |  |  |  |  |  |  | ✓ | ✓ |  |  |  |  |  |
| (SERIS, 2020) | SERIS 2020 | NR | Update of the Solar PV Roadmap for Singapore | Singapore | 2020 |  | ✓ |  |  |  |  |  |  |  | ✓ |  |  |  |  |  |
| (Chaianong et al., 2020) | Chaianong et al. 2020 | J | Customer economics of residential PV–battery systems in Thailand | Thailand | 2020 |  |  |  |  |  |  |  |  |  | ✓ |  |  |  |  |  |
| (Mongird et al., 2020) | Mongird et al. 2020 | J | An evaluation of energy storage cost and performance characteristics | U.S. | 2020 |  |  |  |  |  | ✓ |  |  |  |  |  |  |  |  |  |
| (He et al., 2020) | He et al. 2020 | J | Greenhouse gas consequences of the China dual credit policy | China | 2020 |  |  |  |  |  | ✓ |  |  |  |  |  |  |  |  |  |
| (Vartiainen et al., 2020) | Vartiainen et al. 2020 | J | Impact of weighted average cost of capital, capital expenditure, and other parameters on future utility-scale PV levelised cost of electricity | Europe | 2020 | ✓ |  |  |  |  | ✓ | ✓ |  |  |  |  |  |  | ✓ |  |
| (Deorah et al., 2020) | Deorah et al. 2020 | GR | Estimating the Cost of Grid-Scale Lithium-Ion Battery Storage in India Energy Technologies Area Lawrence Berkeley National Laboratory | India | 2020 |  |  |  |  |  | ✓ |  |  |  |  |  |  |  |  |  |
| (Resch et al., 2020) | Resch et al. 2020 | J | Market uptake of concentrating solar power in Europe: model-based analysis of drivers and policy trade-offs Deliverable 8.2 | Europe | 2020 |  |  |  |  |  |  |  |  |  |  | ✓ |  |  |  |  |
| (Mahone et al., 2020) | Mahone et al. 2020 | GR | Hydrogen Opportunities in a Low-Carbon Future An Assessment Of Long-Term Market Potential in the Western United States | U.S. | 2020 |  |  |  |  |  |  | ✓ | ✓ |  |  |  |  |  | ✓ | ✓ |
| (Hall et al., 2020) | Hall et al. 2020 | NR | The Potential Role of Hydrogen in India: A pathway for scaling-up low carbon hydrogen across the economy | India | 2020 |  |  |  |  |  |  | ✓ | ✓ |  |  |  |  |  | ✓ | ✓ |
| (BNEF, 2020) | BNEF 2020 | CR | Hydrogen Economy Outlook | China | 2020 |  |  |  |  |  |  | ✓ |  |  |  |  |  |  | ✓ |  |
| (Gallardo et al., 2021) | Gallardo et al. 2021 | J | A Techno-Economic Analysis of solar hydrogen production by electrolysis in the north of Chile and the case of exportation from Atacama Desert to Japan | Chile | 2020 |  |  |  |  |  |  |  | ✓ |  |  |  |  |  |  | ✓ |
| (Peterson et al., 2020) | Peterson et al. 2020 | GR | DOE Hydrogen and Fuel Cells Program Record: Hydrogen Production Cost From PEM Electrolysis | U.S. | 2020 |  |  |  |  |  |  |  | ✓ |  |  |  |  |  |  | ✓ |
| (OEDI, 2020) | OEDI 2020 | GR | 2020 Annual Technology Baseline (ATB) Cost and Performance Data for Electricity Generation Technologies | U.S. | 2020 | ✓ | ✓ | ✓ | ✓ | ✓ |  |  |  | ✓ | ✓ | ✓ | ✓ | ✓ |  |  |
| (IEA, 2020) | IEA 2020 | NR | World Energy Outlook 2020 | U.S., Europe, China, India | 2020 | ✓ |  |  | ✓ | ✓ | ✓ | ✓ | | ✓ |  |  | ✓ | ✓ |  |  |
| (OEDI, 2019) | OEDI 2019 | GR | 2019 Annual Technology Baseline (ATB) Cost and Performance Data for Electricity Generation Technologies | U.S. | 2019 | ✓ | ✓ | ✓ | ✓ | ✓ |  |  |  | ✓ | ✓ | ✓ | ✓ | ✓ |  |  |
| (ETIP, 2019) | ETIP 2019 | NR | Fact sheets about photovoltaics (PV) - The cost of PV systems | Europe (Germany, France, Spain, United Kingdom, Italy) | 2019 | ✓ | ✓ |  |  |  |  |  |  | ✓ | ✓ |  |  |  |  |  |
| (Tlili et al., 2019) | Tlili et al. 2019 | J | Hydrogen market penetration feasibility assessment: Mobility and natural gas markets in the US, Europe, China and Japan | Europe, China, Japan | 2019 |  |  |  |  |  |  |  | ✓ |  |  |  |  |  |  | ✓ |
| (ETC, 2019) | ETC 2019 | NR | China 2050: A fully developed rich net-zero economy | China | 2019 |  |  |  |  |  |  |  | ✓ |  |  |  |  |  |  |  |
| (Graham et al., 2018) | Graham et al. 2018 | GR | GenCost 2018 – Final report | Australia | 2018 | ✓ | ✓ | ✓ | ✓ |  | ✓ |  |  | ✓ |  | ✓ | ✓ |  |  |  |
| (OEDI, 2018) | OEDI 2018 | GR | 2018 Annual Technology Baseline (ATB) Cost and Performance Data for Electricity Generation Technologies | U.S. | 2018 | ✓ | ✓ | ✓ | ✓ | ✓ |  |  |  | ✓ | ✓ | ✓ | ✓ | ✓ |  |  |
| (Kost et al., 2018) | Kost et al. 2018 | NR | Levelized Cost of Electricity- Renewable Energy Technologies (Fraunhofer ISE) | Germany | 2018 |  |  |  |  |  |  |  |  | ✓ | ✓ |  | ✓ | ✓ |  |  |
| (Hayward & Graham, 2017) | Hayward & Graham 2017 | GR | Electricity generation technology cost projections | Australia | 2017 | ✓ | ✓ | ✓ | ✓ |  | ✓ |  |  |  |  |  |  |  |  |  |
| (OEDI, 2017) | OEDI 2017 | GR | 2017 Annual Technology Baseline (ATB) Cost and Performance Data for Electricity Generation Technologies | U.S. | 2017 | ✓ | ✓ | ✓ | ✓ | ✓ |  |  |  | ✓ | ✓ | ✓ | ✓ | ✓ |  |  |
| (ETIP, 2017) | ETIP 2017 | NR | Fact sheets about photovoltaics (PV) - The cost of PV systems | Europe (Germany, France, Spain, United Kingdom, Italy) | 2017 | ✓ | ✓ |  |  |  |  |  |  | ✓ | ✓ |  |  |  |  |  |
| (OEDI, 2016) | OEDI 2016 | GR | 2016 Annual Technology Baseline (ATB) Cost and Performance Data for Electricity Generation Technologies | U.S. | 2016 | ✓ | ✓ | ✓ | ✓ | ✓ |  |  |  | ✓ | ✓ | ✓ | ✓ | ✓ |  |  |
| (OEDI, 2015) | OEDI 2015 | GR | 2015 Annual Technology Baseline (ATB) Cost and Performance Data for Electricity Generation Technologies | U.S. | 2015 | ✓ |  | ✓ | ✓ | ✓ |  |  |  | ✓ |  | ✓ | ✓ | ✓ |  |  |
| (Craigen et al., 2015) | Craigen et al. 2015 | GR | Australian Power Generation Technology Report | Australia | 2015 | ✓ | ✓ | ✓ | ✓ |  | ✓ |  |  | ✓ | ✓ | ✓ | ✓ |  |  |  |
| (Kost et al., 2013) | Kost et al. 2013 | NR | Levelized Cost of Electricity- Renewable Energy Technologies (Fraunhofer ISE) | Germany | 2013 |  |  |  |  |  |  |  |  | ✓ | ✓ | ✓ | ✓ | ✓ |  |  |
| (Kost et al., 2012) | Kost et al. 2012 | NR | Levelized Cost of Electricity- Renewable Energy Technologies (Fraunhofer ISE) | Germany, Spain | 2012 |  |  |  |  |  |  |  |  | ✓ | ✓ | ✓ | ✓ | ✓ |  |  |
| (DNV, 2023) | DNV 2023 | NR | Energy Transition Outlook 2023: A global and regional forecast to 2050 | World | 2023 |  |  |  |  |  |  |  |  | ✓ |  |  | ✓ |  |  |  |
| (Abbott et al., 2023) | Abbott et al. 2023 | J | Accelerating the Renewable Energy Revolution to Get Back to the Holocene | World | 2023 |  |  |  |  |  | ✓ |  |  | ✓ |  |  | ✓ |  |  |  |
| (BNEF, 2023) | BNEF 2023 | CR | Lithium-Ion Battery Pack Prices Hit Record Low of $139/kWh | World | 2023 |  |  |  |  |  | ✓ |  |  |  |  |  |  |  |  |  |
| (Rozon et al., 2023) | Rozon et al. 2023 | J | Long-Term Forecasting Framework for Renewable Energy Technologies’ Installed Capacity and Costs for 2050 | World | 2023 |  |  |  |  |  |  |  |  |  |  | ✓ |  |  |  |  |
| (Gordon, 2022) | Gordon 2022 | CR | Battery market forecast to 2030: Pricing, capacity, and supply and demand | World | 2022 |  |  |  |  |  | ✓ |  |  |  |  |  |  |  |  |  |
| (BP, 2022) | BP 2022 | CR | BP Energy Outlook: 2022 Edition | World | 2020 |  |  |  |  |  |  |  |  | ✓ |  |  |  | ✓ |  |  |
| (US DoE, 2022) | US DoE 2022 | GR | Financial Incentives for Hydrogen and Fuel Cell Projects | World | 2022 |  |  |  |  | ✓ |  |  |  |  |  |  |  |  |  |  |
| (DNV, 2022) | DNV 2022 | NR | Hydrogen Forecast to 2050 Energy Transition Outlook 2022 | World | 2022 |  |  |  |  |  |  | ✓ | ✓ |  |  |  |  |  | ✓ |  |
| (Jacobson et al., 2022) | Jacobson et al. 2022 | J | Low-cost solutions to global warming, air pollution, and energy insecurity for 145 countries | World | 2022 | ✓ | ✓ |  | ✓ | ✓ |  |  |  | ✓ | ✓ |  | ✓ | ✓ |  |  |
| (Reksten et al., 2022) | Reksten et al. 2022 | J | Projecting the future cost of PEM and alkaline water electrolysers; a CAPEX model including electrolyser plant size and technology development | World | 2022 |  |  |  |  |  |  |  | ✓ |  |  |  |  |  |  |  |
| (IEA, 2022a) | IEA 2022 – H2 | NR | Global Hydrogen Review | World | 2022 |  |  |  |  |  |  |  | ✓ |  |  |  |  |  |  |  |
| (Teske et al., 2021) | Teske et al. 2021 | J | It Is Still Possible to Achieve the Paris Climate Agreement: Regional, Sectoral, and Land-Use Pathways | World | 2021 | ✓ |  |  | ✓ | ✓ |  |  |  |  |  |  |  |  |  |  |
| (Grant et al., 2021) | Grant et al. 2021 | J | Cost reductions in renewables can substantially erode the value of carbon capture and storage in mitigation pathways | World | 2021 | ✓ |  |  | ✓ | ✓ |  |  |  |  |  |  |  |  |  |  |
| (Bogdanov et al., 2021) | Bogdanov et al. 2021 | J | Full energy sector transition towards 100% renewable energy supply: Integrating power, heat, transport and industry sectors including desalination | World | 2021 | ✓ | ✓ |  | ✓ | ✓ | ✓ |  |  |  |  |  |  |  |  |  |
| (Brändle et al., 2021) | Brändle et al. 2021 | J | Estimating long-term global supply costs for low-carbon hydrogen (Base and Optimal scenarios) | World | 2021 |  |  |  | ✓ |  |  |  | ✓ |  |  |  |  |  |  |  |
| (Wiser et al., 2021) | Wiser et al. 2021 | J | Expert elicitation survey predicts 37% to 49% declines in wind energy costs by 2050 | World | 2021 |  |  |  |  |  |  |  |  |  |  |  | ✓ | ✓ |  |  |
| (BNEF, 2021) | BNEF 2021 | CR | New Energy Outlook 2021 | World | 2021 |  |  |  |  |  |  |  |  |  |  |  |  | ✓ |  |  |
| (Mauler et al., 2021) | Mauler et al. 2021 | J | Battery cost forecasting: A review of methods and results with an outlook to 2050 | World | 2021 |  |  |  |  |  | ✓ |  |  |  |  |  |  |  |  |  |
| (WEC, 2021) | WEC 2021 | NR | Decarbonised hydrogen imports into the European Union: challenges and opportunities | World | 2021 |  |  |  |  |  |  |  | ✓ |  |  |  |  |  |  |  |
| (EPRI, 2020) | EPRI 2020 | NR | Battery Energy Storage Lifecycle Cost Assessment Summary | World | 2020 |  |  |  |  |  | ✓ |  |  |  |  |  |  |  |  |  |
| (BNEF, 2020) | BNEF, 2020 | CR | Hydrogen Economy Outlook | World | 2020 |  |  |  |  |  |  |  |  |  |  |  |  |  |  | ✓ |
| (Beuse et al., 2020) | Beuse et al. 2020 | J | Projecting the Competition between Energy-Storage Technologies in the Electricity Sector | World | 2020 |  |  |  |  |  | ✓ |  |  |  |  |  |  |  |  |  |
| (Penisa et al., 2020) | Penisa et al. 2020 | J | Projecting the price of lithium-ion NMC battery packs using a multifactor learning curve model | World | 2020 |  |  |  |  |  | ✓ |  |  |  |  |  |  |  |  |  |
| (IRENA, 2020) | IRENA 2020 | NR | Hydrogen: a Renewable Energy Perspective | World | 2020 |  |  |  |  |  |  | ✓ |  |  |  |  |  |  | ✓ |  |
| (IEA, 2019a) | IEA 2019a | NR | The Future of Hydrogen - Seizing today’s opportunities | World | 2019 |  |  |  |  |  |  |  |  |  |  |  |  |  |  | ✓ |
| (IEA, 2019) | IEA 2019 | NR | World Energy Outlook 2019 | World | 2019 | ✓ |  |  | ✓ | ✓ |  |  |  | ✓ |  |  | ✓ | ✓ |  |  |
| (IEA, 2018) | IEA 2018 | NR | World Energy Outlook 2018 | World | 2018 | ✓ |  |  | ✓ | ✓ |  |  |  | ✓ |  |  | ✓ | ✓ |  |  |
| (IEA, 2017) | IEA 2017 | NR | World Energy Outlook 2017 | World | 2017 | ✓ |  |  | ✓ |  |  |  |  |  |  |  |  |  |  |  |
| (IEA, 2016) | IEA 2016 | NR | World Energy Outlook 2016 | World | 2016 | ✓ |  |  | ✓ | ✓ |  |  |  |  |  |  |  |  |  |  |
| (IEA, 2015) | IEA 2015 | NR | World Energy Outlook 2015 | World | 2015 | ✓ |  |  | ✓ |  |  |  |  |  |  |  |  |  |  |  |
| (IEA, 2014) | IEA 2014 | NR | World Energy Outlook 2014 | World | 2014 | ✓ |  |  | ✓ |  |  |  |  |  |  |  |  |  |  |  |
| (IEA, 2013) | IEA 2013 | NR | World Energy Outlook 2013 | World | 2013 | ✓ |  |  | ✓ | ✓ |  |  |  |  |  |  |  |  |  |  |
| (IEA, 2012) | IEA 2012 | NR | World Energy Outlook 2012 | World | 2012 | ✓ |  |  | ✓ |  |  |  |  |  |  |  |  |  |  |  |
| (IEA, 2011) | IEA 2011 | NR | World Energy Outlook 2011 | World | 2011 | ✓ |  |  | ✓ |  |  |  |  |  |  |  |  |  |  |  |
| (IEA, 2010) | IEA 2010 | NR | World Energy Outlook 2010 | World | 2010 | ✓ |  |  | ✓ |  |  |  |  |  |  |  |  |  |  |  |
| (IEA, 2009) | IEA 2009 | NR | World Energy Outlook 2009 | World | 2009 | ✓ |  |  | ✓ | ✓ |  |  |  |  |  |  |  |  |  |  |
| (IEA, 2008) | IEA 2008 | NR | World Energy Outlook 2008 | World | 2008 | ✓ |  |  | ✓ | ✓ |  |  |  |  |  |  |  |  |  |  |
| (IEA, 2006) | IEA 2006 | NR | World Energy Outlook 2006 | World | 2006 | ✓ |  |  | ✓ | ✓ |  |  |  |  |  |  |  |  |  |  |
| (IEA, 2004) | IEA 2004 | NR | World Energy Outlook 2004 | World | 2004 | ✓ |  |  | ✓ | ✓ |  |  |  |  |  |  |  |  |  |  |
| (IEA, 2003) | IEA 2003 | NR | World Energy Outlook 2003 | World | 2003 | ✓ |  |  | ✓ | ✓ |  |  |  |  |  |  |  |  |  |  |
| (IEA, 2002) | IEA 2002 | NR | World Energy Outlook 2002 | World | 2002 | ✓ |  |  | ✓ | ✓ |  |  |  |  |  |  |  |  |  |  |
| (IEA, 2001) | IEA 2001 | NR | World Energy Outlook 2001 | World | 2001 | ✓ |  |  | ✓ | ✓ |  |  |  |  |  |  |  |  |  |  |
